# An RNA vaccine against adrenomedullin reduces angiogenesis and tumor burden in a syngeneic metastatic melanoma mouse model

**DOI:** 10.3389/fimmu.2025.1604156

**Published:** 2025-06-05

**Authors:** Srdan Tadic, Laura Ochoa-Callejero, Judit Narro-Íñiguez, Josune García-Sanmartín, Alfredo Martínez

**Affiliations:** Angiogenesis Unit, Oncology Area, Center for Biomedical Research of La Rioja (CIBIR), Logroño, Spain

**Keywords:** adrenomedullin, angiogenesis, mRNA vaccine, melanoma, lung metastases, lipid nanoparticles

## Abstract

**Introduction:**

Adrenomedullin (AM) is an autocrine/paracrine growth factor as well as a crucial regulator of angiogenesis and immune response. AM is overexpressed by most solid tumors, which makes it a good target for anti-tumor therapy.

**Methods:**

In this study, we designed and tested an mRNA vaccine directed against a fusion antigen composed by a small piece of keyhole limpet hemocyanin (KLH), as a hapten, and mouse AM. The *in vitro*-synthesized mRNA was encapsulated in lipid nanoparticles (LNPs) and injected in C57BL/6 mice. Empty LNPs were used as a negative control. After five immunizations, B16-F10 melanoma cells were injected through the tail vein to induce lung metastases. In addition, transgenic mice expressing green fluorescent protein in the blood vessels (SMAA-GFP) were also immunized with both LNP types to assess the potential side-effects of the vaccine on normal blood vessels.

**Results:**

Antibody titers against AM and the number of CD8^+^ T cells were significantly higher in AM-immunized mice than in negative controls. Furthermore, the number and size of the lung metastases, as well as the number of blood vessels, were significantly reduced in the AM-immunized group. In addition, no significant differences were observed in the number and distribution of existing blood vessels after immunization of the SMAA-GFP animals.

**Discussion:**

In summary, we have shown that an mRNA vaccine against the fusion KLH-AM peptide was able to break peripheral immunotolerance and induce a specific response against the angiogenic factor AM thus reducing tumor burden in the absence of disturbances to normal blood vessels.

## 1 Introduction

Cancer is currently the leading cause of death in the world, with about 10 million people estimated to have died from this disease in 2022. The most lethal cancer types were the ones affecting the lung, colon, liver, stomach and breast (1). Unique features of this disease, the so-called hallmarks of cancer, explain cancer cells´ ability to propagate, survive, grow, invade and metastasize (2). Metastasis is one of the most prominent and complex cancer hallmarks and it represents the ability of some cancer cells to invade and colonize a distant tissue or organ. Metastatic disease is responsible for more than 90% of deaths among cancer patients (3). Therefore, metastatic disease is the number one cancer-related health emergency issue worldwide that needs an adequate solution. One of the crucial processes required for metastasis initiation, as well as for tumor growth, is angiogenesis (4).

Angiogenesis implies the formation of new blood vessels from established vascular beds with the help of endothelial and stromal cells, and represents another cancer hallmark. Tumor growth and metastasis are high energy demanding processes, so they require increased nutrient and oxygen availability and therefore high vascularity (5). Furthermore, solid tumors become hypoxic as they grow from a small seed to larger masses, thus requiring a denser vascular system to support their needs (6). Beside energy and nutrients, tumor-related vasculature provides conditions for tumor cells to metastasize and evade the immune response. The process of angiogenesis is regulated by a complex interaction between pro-angiogenic and anti-angiogenic factors (7). Under normal physiological conditions, a balance between pro- and anti- angiogenic factors is reached, thus the vasculature of most adult tissues is relatively quiescent and stable (8). However, under pathological conditions, such as cancer, the situation is drastically different and pro-angiogenic signaling is dominant (9). Therefore, tumor-related angiogenesis and pro-angiogenic factors represent important targets for anti-tumor therapies.

Angiogenesis is triggered by hypoxia and usually mediated by the HIF1 system (10). HIF1 is a heterodimeric transcription factor that binds specific sequences (hypoxia response elements, HREs) in the promotor region of survival and pro-angiogenic genes, such as VEGF (11) and adrenomedullin (AM) (12). AM is a 52 amino acid peptide in humans (50 amino acids in rodents) that belongs to the calcitonin/calcitonin gene-related peptide family and is a crucial pro-angiogenic factor (13). AM is synthesized as part of a larger precursor molecule that consists of 185 amino acids, called preproadrenomedullin. AM exerts its actions through specific receptors, which are a combination of the calcitonin receptor like receptor (CLR) and either receptor activity-modifying protein 2 (RAMP2) or RAMP3 (known as AM_1_ and AM_2_ receptors, respectively) (14).

AM acts as an autocrine/paracrine growth factor for tumor cells (15), it promotes motility and metastasis of tumor cells, prevents tumor cell death by apoptosis and supports angiogenesis and tumor cell survival while promoting tumor evasion mechanisms (16). AM´s expression is highly up-regulated in most solid tumors, which makes it a good target for anti-tumor therapy. Different studies confirmed that targeting AM or its receptors has a direct impact on tumor size, progression of the disease and patient’s survival (17).

Different approaches have been developed to clinically target pro-angiogenic factors and receptors, such as targeted-therapy and chemotherapy (18). Although relatively standard, these therapies are still having limited success and therefore there is a need for the development of new therapeutic approaches (19, 20). Cancer vaccines are an attractive approach since they can initiate the body’s own immune response against tumor-related antigens that may lead into tumor´s reduction/destruction and prevention of recurrence (21). Tumor angiogenesis represents a promising target for cancer vaccines due to the high genetic stability of endothelial cells, target overexpression limited to tumor tissue, rare occurrence in normal adult tissues, and broad presence of the target in different tumor types (22, 23). Cancer vaccines based on nucleic acids, with emphasis on mRNA, have several advantages over more classic vaccination approaches including high efficiency, simplicity, low cost and rapid adaptability and thus they represent a highly promising avenue in cancer therapeutics development, with numerous clinical trials currently ongoing (24). Many DNA cancer vaccines have been developed against angiogenic molecules with relative success in animal models while some of them have reached clinical trials (25). Interestingly, in many cases, the antiangiogenic vaccines did not interfere with extra-tumoral vasculature, lowering the main theoretical caveat elicited by the use of such vaccines (25). On the other hand, we cannot find in the literature examples of mRNA cancer vaccines directed against angiogenic targets.

Considering that pro-angiogenic factors represent self-antigens, directing the immune response against these peptides requires overcoming the immune tolerance mechanisms. This can be achieved with the help of foreign antigens (haptens) such as keyhole limpet hemocyanin (KLH) or other immune response boosters with adjuvant capability (26, 27).

Our goal in this study was to demonstrate the feasibility and efficacy of an mRNA vaccine targeting an angiogenic self-antigen. To accomplish this, we designed an mRNA expressing a fusion protein made up of a small fragment of KLH linked to mouse AM. This mRNA was encapsulated into LNPs and injected into C57BL/6 mice that were eventually challenged with syngeneic melanoma cells following a metastatic lung tumor model.

## 2 Materials and methods

### 2.1 Selection of the optimal KLH fragment and design of the fusion protein (antigen)

The sequences of KLH1 and of murine AM were obtained from the Universal Protein Database (UniProt). Sequences were reverse translated to the proper DNA sequence by using BCCM/GeneCorner (https://www.genecorner.ugent.be/https://www.genecorner.ugent.be/rev_trans.html) online tool.

The KLH1 protein functional sub-units (FU) a, b, c, d, e, f, g and h were analyzed looking for B cell and T cell epitope binding and immunogenicity prediction using the free epitope database and prediction resource (IEDB; http://www.iedb.org), as performed before (28, 29). Other free algorithms used to characterize KLH1 FUs were Bepipred for presence of B-linear epitopes, Kolaskar and Tongaonkar for immunogenicity prediction, Chou and Fasman for Beta Turn prediction, Karplus and Schulz for flexibility prediction, Parker for hydrophilicity prediction, and Emini for Surface Accessibility prediction. Finally, IEDB server Discotope portal was used for prediction of conformational epitopes on KLH1 protein sub-units (**Supplementary Figure S1**).

The KLH1 FUs were further analyzed for the presence of epitopes that can bind to MHC I and II (HLA) complexes/alleles by using IEDB server MHC binding prediction portal. For MHC I, mouse alleles included in the prediction were H-2Db, H-2Dd, H-2Kb, H-2Kd, H-2Kk and H-2Ld. The prediction method used was NetMHCpan EL 4.1. and binding capacity was evaluated based on score value, where a low score correlates with higher binding capacity. MHC II mouse alleles included H-2IAb, H-2IAd and H-2IEd and the consensus method was applied for prediction. The method is based on the rank values and lower values correlate with better binders. Additionally, prediction of MHCI-KLH1 sub-unit peptide complex immunogenicity was performed using IEDB server MHCI immunogenicity prediction tool (**Supplementary Figure S2**).

Based on the scores obtained in all analyses, the KLH1 FU with the highest scores was further evaluated. Different sequence fragments of this FU were analyzed using all methods described above in order to find the optimal fragment to be bound to mouse AM, looking for the highest scores and the minimum size of the fragment. The chosen fragment´s DNA sequence was then bound to mouse AM´s sequence to code for the fusion protein that was to constitute the vaccine´s antigen.

### 2.2 Codon optimization and construction of the expression vector

**Figure 1:**
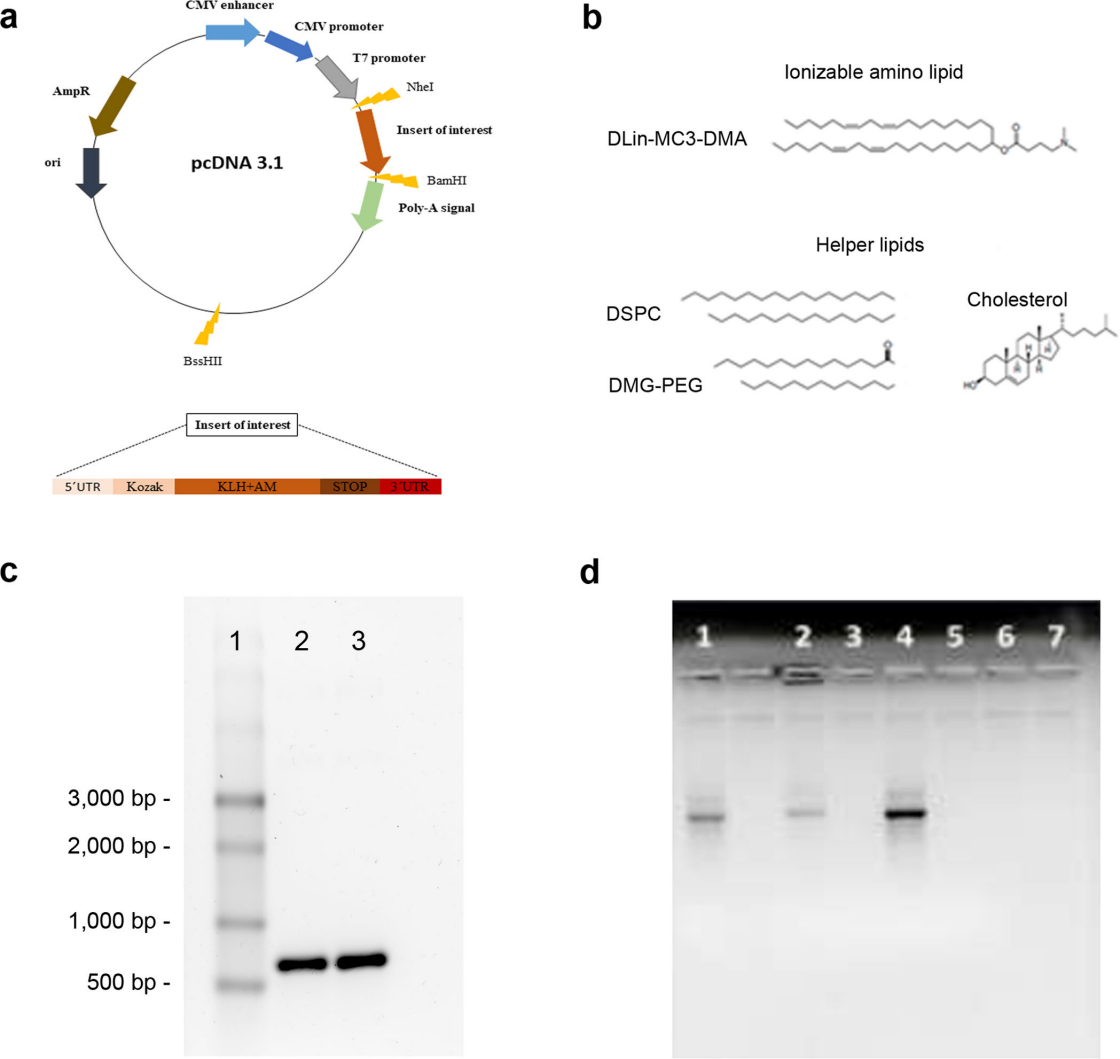
Vaccine construction. A synthetic DNA containing all the vaccine components was inserted into the pcDNA 3.1 vector **(a)**. The lipids used in the generation of the LNPs are shown **(b)**. The KLH-AM mRNA before (2) and after (3) capping was characterized in an agarose gel. A single stranded RNA ladder (1) was added for size reference **(c)**. Encapsulation efficiency was investigated by gel electrophoresis of naked mRNA (1), non-solubilized mRNA-containing LNPs (2), non-solubilized empty LNPs (3), solubilized mRNA-containing LNPs (4), solubilized empty LNPs (5), flow-through of mRNA-containing LNPs (6) and flow-through of empty LNPs (7) **(d)**.

Besides the open reading frame (ORF) for the fusion protein, the final expression construct was complemented with the insertion of a typical 3’untranslated region (3’UTR) and a 5’untranslated region (5’UTR) that are necessary for mRNA synthesis (**Table 1**). In addition, a standard Kozak sequence (gccaccatg) and stop codon (tag) were added. The final sequence was optimized by using Integrated DNA Technologies (IDT, eu.idtdna.com), Benchling (www.benchling.com) and GenScript (www.genscript.com) free sequence/codon optimization tools. The optimization was performed aiming to reduce rare codon presence, rebalance codon usage with emphasis on GC couples, decrease complexity, decrease secondary structure formation and uridine depletion possibilities. The sequence was built by gene synthesis and inserted into the pcDNA 3.1 plasmid by GenScript Biotech (Piscataway, NJ, USA) downstream of the T7 promoter (**Figure 1a**).

**Table 1:** DNA sequences used for the construction of the KLH-AM construct.

Name	Sequence
5´UTR	gctagcggggaaataagagagaaaagaagagtaagaagaaatataaga
Kozak	gccaccatg
KLH ORF	ccttactgggatggaacgactgcctttacagctttgcccacctttgtgacagatgaagaggac aaccctttccaccatggtcacattgactaccttggtgtggacaccacacgaagtccacggg acaaactctttaatgatccagagagagggtcagagtccttcttctatagacaagttctactg gctctggacagacagacttctgccagttcgaagtccagtttgaaataactcat
AM ORF	tataggcaaagcatgaaccaaggatctcggagcaatggctgtagatttgggacctgcac cttccagaagctggcccaccagatctaccagctcactgataaagacaaggatggcatgg caccgaggaacaagatctccccccagggctatcgtcggcgccgcaggtgagctgccttc tgcggggcttgccttctggccatgcccttcttctctcccttgcacctgtacctcttggtctttg aataaagcctgagtaggaaggcggccgctcgagcatgcatctagaaaggatcc
3´UTR	gctgccttctgcggggcttgccttctggccatgcccttcttctctcccttgcacctgtacctct tggtctttgaataaagcctgagtaggaaggcggccgctcgagcatgcatctagaaaggatcc
Full sequence	gctagcggggaaataagagagaaaagaagagtaagaagaaatataagagccaccatgc cttactgggatggaacgactgcctttacagctttgcccacctttgtgacagatgaagaggac aaccctttccaccatggtcacattgactaccttggtgtggacaccacacgaagtccacggg acaaactctttaatgatccagagagagggtcagagtccttcttctatagacaagttctactg gctctggagcagacagacttctgccagttcgaagtccagtttgaaataactcattataggca aagcatgaaccaaggatctcggagcaatggctgtagatttgggacctgcaccttccagaa gctggcccaccagatctaccagctcactgataaagacaaggatggcatggcaccgaggaa caagatctccccccagggctatcgtcggcgccgcaggtgagctgccttctgcggggcttg ccttctggccatgcccttcttctctcccttgcacctgtacctcttggtctttgaataaagcctg agtaggaaggcggccgctcgagcatgcatctagaaaggatcc

### 2.3 Transfection of *E. coli*, amplification and linearization of DNA template

The expression vector was inserted into competent *E. coli* following standard heat shock protocols and selection in ampicillin plates. The vector was purified using Plasmid DNA MaxiPrep kit (Qiagen, Hilden, Germany) following manufacturer´s instructions. The DNA template was linearized using BssHII restriction enzyme (NewEngland BioLabs, Ipswich, UK) and purified by isopropanol precipitation. The linearized template was resuspended in HyClone HighPure water (Cytiva, Marlborough, MA, USA).

### 2.4 *In vitro* transcription, capping and characterization

The therapeutic mRNA was synthesized by *in vitro* transcription (IVT) using T7-Scribe™ Standard RNA IVT Kit (CellScript, Madison, WI, USA) to produce standard/non-modified mRNA. Alternatively, for *in vivo* experiments, modified mRNA was generated by using the INCOGNITO™ T7 5mC- & Ψ-RNA Transcription Kit (CellScript), following manufacturer´s instructions. Briefly, the linearized DNA template was mixed with components of the kit and reaction incubated at 37°C for 2h. Then, DNAse I was added to remove traces of the DNA template. The reaction product was purified by standard ammonium acetate precipitation, and dissolved in RNAse-free water. The amount and purity of the RNA were analyzed on a NanoDrop spectrophotometer (Thermo Fisher Scientific, Waltham, MA, USA).

Capping of modified IVT-mRNA was done post-transcriptionally using the ScriptCap™ Cap 1 Capping System (CellScript). The Cap1 structure was added by using both ScriptCap Capping Enzyme, and ScriptCap 2’-O-Methyltransferase, according to manufacturer’s instructions. The reaction was performed at 37°C for 2h. The resulting capped mRNA was further characterized by electrophoresis.

### 2.5 Encapsulation of the mRNA in lipid nanoparticles

LNPs were prepared using the ionizable lipid, 4-(dimethylamino)-butanoic acid, (10Z,13Z)-1-(9Z,12Z)-9,12-octadecadien-1-yl-10,13 nonadecadien-1-yl ester (DLin-MC3-DMA, Cayman Chemical, Ann Arbor, MI, USA). In addition, three helper lipids were added, including disteraroylphosphatidylcholine (DSPC, Avanti Polar Lipids, Alabaster, AL, USA), 1,2-dimyristoyl-rac-glycero-3-methoxypolyethylene glycol (DMG-PEG 2000, Cayman Chemical), and cholesterol (Thermo Fisher Scientific) (**Figure 1b**). To generate the particles, the lipids were dissolved in ethanol whereas the mRNA was dissolved in citrate buffer (pH 4.0). These solutions were mixed at a 1:3 volume ratio, to achieve a 40:1 weight lipid/mRNA ratio, by rapid pippeting (30). Constructs were allowed to stabilize for 15 minutes and then were transferred to Amicon Ultrafilter tubes, where they were centrifuged at 13,000 x g, at 4°C, for 30 min. The flow-through was kept for subsequent analyses, whereas the solution remaining inside the tubes was transferred to fresh tubes and centrifuged at 13,000 x g, at 4°C, for 2 min. Nanoparticles where in the pellet of the last spin. Control LNPs were prepared following the same protocol in the absence of mRNA.

### 2.6 Characterization of lipid nanoparticles

Size, polydispersity (PDI) and zeta potential of LNPs encapsulating or not (control) mRNA were analyzed with Zetasizer (Malvern, Worcestershire, UK) according to manufacturer´s instructions. Briefly, samples were diluted in PBS and injected into specialized capillaries. Samples were transferred to standard cuvettes and their size and PDI measured. Six measurements were taken per sample and the mean ± SD was calculated.

To determine encapsulation efficiency, LNPs (both empty and encapsulating mRNA) were separated in two groups; LNPs in Group 1 were solubilized with 2% Triton X-100 and Tris-EDTA and heated at 37°C for 10 min while shaking. On the other hand, LNPs in Group 2 were not solubilized. Naked mRNA was used as a control. Finally, all samples were electrophoresed in 1.0% agarose gels and images recorded with a gel imaging system (BioRad, Hercules, CA, USA).

Furthermore, fluorimetric measurements were performed by using a high sensitivity kit for detection of RNA (HS RNA, Invitrogen, Carlsbad, CA, USA) and a Qubit fluorometer (Thermo Fisher Scientific) following manufacturer´s instructions. Final calculations were done based on the amount of mRNA present in solubilized samples divided by the initial mRNA used for encapsulation and multiplied by 100, with the formula:


mRNAfinal= (mRNAsample/mRNAinitial) x 100

### 2.7 Cell lines and culture conditions

Murine cell lines RAW 264.7 (RRID CVCL_0493, macrophages), B16-F10 (RRID CVCL_0159, melanoma) and PC12 (RRID CVCL_0481, pheochromocytoma) were obtained from the American Type Culture Collection (ATCC, Manassas, VA, USA). They were cultured in Roswell Park Memorial Institute (RPMI 1640, Corning, Manassas, VA, USA), for B16-F10, or in Dulbecco´s Modified Eagle´s Medium (DMEM, Corning) for the other cell lines; all of them supplemented with 10% fetal bovine serum (FBS, Thermo Fisher Scientific), at 37°C and 5% CO_2_ in a humidified incubator. Cell lines were authenticated by STR profiling (IDEXX BioAnalytics, Kornwestheim, Germany).

### 2.8 Exposure of RAW 264.7 cells to LNPs containing fluorescent mRNA

The KLH-AM mRNA was labeled with the HighYield T7 Cy5 RNA Labeling Kit (Jena Bioscience, Jena, Germany), following manufacturer´s instructions. The Cy5-labeled mRNA, as well as unlabeled mRNA as a control, were encapsulated into LNPs. RAW 264.7 cells were seeded into 2-well chambered coverslips (Ibidi, Gräfelfing, Germany) and incubated until reaching 70-90% confluence. Then the LNPs encapsulating the labeled (or not) mRNAs were added. After 2 h, cells were washed thrice with PBS and incubated in fresh media. Cells were observed in an LSM 980 confocal microscope (Zeiss, Oberkochen, Germany) and live imaging was performed.

### 2.9 Animals and immunization protocol

All experiments and animal procedures were carried out following the guidelines laid down by the European Parliament and Counsel (2010/63/UE) and Spanish Royal Decree 53/2013 on animal experimentation, and were revised and approved by the Committee for Animal Welfare of the Center for Biomedical Research of La Rioja (OEBA/CIBIR), Spain, ref. AMR-17.

Immunocompetent mice (7-week-old male C57BL/6 mice) were purchased from Inotiv (Indianapolis, IN, USA). In addition, transgenic mice that express green fluorescent protein (GFP) under the alpha smooth muscle actin (αSMA) promoter, also of C57BL/6 genetic background, were used to investigate vaccine effects on the existing vasculature. These mice (SMAA-GFP) were generated at the National Eye Institute (NEI, NIH, Bethesda, MD, USA) (31) and have been used mainly to study angiogenesis in the context of ophthalmological diseases (32). These animals were a generous gift from late Prof. John Paul Sangiovanni (NEI).

Animals were housed under specific pathogen-free conditions at the CIBIR animal facility (ES260890000992), in a temperature-controlled room with 12-h light/dark cycle and reared on standard chow and water provided *ad libitum*. All animals were checked by a veterinarian, and weighed, on a weekly basis, to ensure animal wellbeing and lack of vaccine toxicity.

For immunization, C57BL/6 mice were randomly divided into two experimental groups: control (n=10) and treated (n=10). The control group received empty LNPs, while the treated group received LNPs containing the KLH-AM mRNA. Animals were injected intramuscularly (i.m.) in the rear hind muscle with 50 µl of the nanovaccine containing a total of 5.0 µg of mRNA (or with empty LNPs for the control). Five immunizations were implemented at two-week intervals, with the last injection given 3 days after intravenous (i.v.) tumor challenge with B16-F10 cells. Animals were sacrificed 3 weeks after tumor challenge and blood and tissues were collected for further analysis.

Furthermore, eighth male SMAA-GFP mice were used. As with the previous group, these animals were randomly divided into two experimental groups: control (n=4) and treated (n=4) and subjected to five immunizations with either control (empty LNPs) or with LNPs containing the KLH-AM mRNA. Three weeks after the final immunization, all animals were sacrificed. After sacrifice, fresh highly vascularized tissues were photographed under UV-light (DM6000B microscope, Leica, Wetzlar, Germany) to assess potential side effects of the vaccine on existing blood vessels.

### 2.10 Tumor challenge

To test the antitumor effects of the vaccine, immunized animals were challenged with syngeneic B16-F10 melanoma tumor cells. For the metastasis model, mice were inoculated i.v. through the tail vein with 2 x 10^5^ B16-F10 cells/animal in order to induce lung metastases, as reported (33). Three weeks after tumor challenge, mice were sacrificed, blood was taken for antibody titration, spleens were collected for lymphocyte analysis and the number and size of metastases was assessed on the lungs.

### 2.11 Morphological and microscopical assessment of resulting tumors

After dissection, lungs were weighed, photographed and the number of visible metastases was recorded. Additionally, the size of the largest metastatic node was determined with a digital caliper for each animal. After that, the left pulmonary lobe was snap frozen in liquid nitrogen and stored for molecular analyses. The remaining lobes were insufflated with 10% formalin, fixed for 24h, and paraffin embedded. Tissue sections (3 µm-thick) were stained with hematoxylin and eosin and the number of metastases per section was assessed under a light microscope (DM6000B, Leica).

### 2.12 Immunohistochemistry

Additional sections were rehydrated and antigen retrieval was performed by heating in citrate buffer (pH 6.0) for 20 min at 96°C. After blocking with normal donkey serum, sections were incubated overnight with primary antibodies including anti-CD31 and anti-Ki67 (**Table 2**). The next day, following several washes in PBS, sections were incubated with a polymer complex containing HRP (RE7200-CE, Leica, RRID AB_3674357). Immunoreactivity was developed with diaminobenzidine (DAB, Dako). Slides were lightly counterstained with hematoxylin. Pictures were taken in random fields (n=10 per mouse) and the area occupied by the blood vessels (for CD31) or the number of positive cells (Ki67) per mm^2^ was quantified through free digital pathology QuPath software (34).

**Table 2:** .Antibodies used in the study.

Assay	Antibody	Manufacturer	Cat. No.	RRID	Dilution
Imaging	Anti-Mouse IgG Alexa Fluor™ Plus 647	Thermo Fisher	A32787	AB_2762830	1/5,000
ELISA	Anti-mouse IgG HRP	Jackson ImmunoResearch, West Grove, PA, USA	715-035-151	AB_2340771	1/10,000
Flow cytometry	Anti-mouse CD8b.2 FITC	BioLegend, San Diego, CA, USA	140404	AB_10643587	1/100
	Anti-mouse CD4 APC	BD, Franklin Lakes, NJ, USA	561859	AB_10894581	1/100
	Anti-mouse CD4 APC Cy7	BioLegend	100526	AB_312727	1/100
IHC	Anti-mouse CD31	Abcam, Cambridge, UK	ab281583	AB_3096925	1/5,000
	Anti-mouse Ki67 (clone SP6)	Vitro Master Diagnostica, New York, NY, USA	MAD-000310QD-3	AB_3677420	1/5

### 2.13 Serum characterization (ELISA and immunocytochemistry)

Sera was isolated from blood following standard protocols. For ELISA, COSTAR high-binding 96-well plates (Corning, New York, NY, US) were coated with 100 ng/50 µL/well of mouse AM peptide (Phoenix Pharmaceuticals, Burlingam, CA, USA), dissolved in carbonate coating buffer pH 9.6, and incubated overnight at 4°C. The next day, plates were washed with PBS and then blocked with 1.5% FBS in PBS for 1h at RT with shaking. Sera samples were serially diluted in PBS (from 1:50 to 1:1,600), added to wells and incubated at RT with shaking for 2h. Wells were washed again and incubated with anti-mouse secondary antibody bound to HRP (**Table 2**) at a 1:10,000 dilution for 1h. After washing again, 3,3′,5,5′-Tetramethylbenzidine (TMB) substrate was added to each well. Color development was stopped with 3N HCl and plates were analyzed on a microplate reader (POLARstar Omega, BMG Labtech, Germany) at 492 nm.

In addition, to ensure that the IgGs identified by ELISA were able to recognize intrinsic cell-produced AM, PC12 cells were used as a control since they have been shown to express high levels of AM (35). Since these cells do not stick strongly to the substrate, they were collected into cytospin slides. Five thousand cells were added to each cytospin funnel and they were centrifuged at 1,500 rpm for 5 min in a Shandon Cytospin 4 (Thermo Fisher Scientific). Then, cells were fixed in 4% paraformaldehyde for 10 min, washed with PBS and permeabilized with 1.0% Triton X-100 in PBS for 10 more min. Slides were blocked with 5.0% donkey serum. Then, cells were incubated overnight at 4°C with either serum from vaccinated animals at a 1/100 dilution or serum isolated from control animals at the same dilution. In addition, other slides were exposed to PBS as a negative control. The next day, cells were washed with PBS and then incubated with secondary anti-mouse IgG AlexaFluor 647 antibody (**Table 2**) and 1/1,000 Hoechst 33342 (Sigma Aldrich) as a nuclear stain, for 1 h at RT, in the dark. Following incubation, cells were washed several times with PBS, slides were mounted and imaging was performed using an SP5 confocal microscope (Leica).

### 2.14 Isolation of splenocytes

Spleens were collected from immunized animals, minced in small pieces and passed through 70 µm cell strainers (Corning). Resulting cells were resuspended in RPMI 1640 media supplemented with 1.0% penicillin/streptomycin (Gibco). After three washes to remove cell debris, cells were exposed to Ammonium–Chloride–Potassium (ACK) red blood cell lysis buffer (Thermo Fisher Scientific) on ice. Then several washes were performed until a clear pellet was obtained. Finally, cells were resuspended in RPMI 1640 media supplemented with 10% FBS and 1.0% penicillin/streptomycin.

### 2.15 Flow cytometry

For cell labeling, aliquots of 1.0 x 10^6^ cells per animal were prepared. Staining was performed for surface markers CD4 and CD8 according to standard protocols. Briefly, cells were resuspended in FACS buffer (2.0% FBS plus 0.01% ammonium azide in PBS). Then, cells were incubated with Fc block (anti-CD16/32, BD, Franklin Lakes, NJ, USA) in FACS buffer for 5 min on ice. Cells were then washed, centrifuged at 1,500 rpm at 4°C, and the supernatant discarded. Cells were then exposed to an antibody mixture of CD4 APC-Cy7 and CD8 FITC (**Table 2**) in FACS buffer and incubated for 30 min on ice, in the dark. Flow cytometry was performed on a FACSCantoII cytometer (BD).

### 2.16 Modified immune-mediated cell killing assay

B16-F10 cells were seeded at 1.0 x 10^4^ cells/well on 96-well plates (Corning) and incubated until reaching 70-90% confluence. Cells were then washed with PBS and splenocytes, isolated from either immunized or control animals, were added in triplicates at a ratio of 1:5 (tumor cells: splenocytes), and incubated for different times in RPMI supplemented with 10% FBS. As controls, wells with only splenocytes or only tumor cells were also used. After this time, the colorimetric CellTiter 96^®^ AQ_ueous_ One Solution Cell Proliferation Assay (MTS, Promega, Madison, WI, USA) was applied following manufacturer’s instructions. Briefly, 20 µL of solution were added to each well, cells were incubated in the dark for 4 h, and then analyzed on the microplate reader (POLARstar Omega) at 490 nm.

### 2.17 Statistics

All data sets were tested for normalcy and homoscedasticity. Normal data were analyzed by Unpaired Student’s t-test or by 2-way ANOVA. Data that did not follow a normal distribution were compared with the Mann-Whitney´s *U* test. All these studies were performed with GraphPad Prism version 5.02 (GraphPad Software, Inc. La Jolla, CA). A p< 0.05 was considered statistically significant.

## 3 Results

### 3.1 Construction and characterization of the vaccine

Mouse AM is a 50-amino acid regulatory peptide that is broadly expressed through mouse tissues and organs (36). Thus, if we want to generate an immune response and break the immune tolerance for this intrinsically generated molecule we need to add a hapten. KLH has been commonly used for this task (27) but this is a huge molecule with a molecular weight ranging from 4,500 to 13,000 kDa, depending on its glycation state and the number of subunits (37). Therefore, we decided to select a small fragment of this sea snail protein with enough immunogenicity, beta turn, flexibility, hydrophobicity and surface accessibility, with help from freely available predictive software (**Supplementary Figures S1**, **S2**). At the end, we selected the sequence that is shown in **Table 1**. Following codon optimization, the final sequence containing a 5´UTR, a Kozak sequence for ribosome engagement, the ORF of the KLH-AM fusion and a 3´UTR (**Table 1**) was built by gene synthesis and inserted into the pcDNA 3.1 plasmid through a service contract with GenScript Biotech. The plasmid provided the T7 RNA polymerase promoter and the polyA tail (**Figure 1a**).

The mRNA was synthesized *in vitro* in the presence of modified nucleotides and the cap was post-transcriptionally added. When the resulting RNAs were run in a gel, the apparent size was close to the expected one at about 750 nucleotides (nt) (**Figure 1c**).

The mature mRNA was encapsulated into LNPs as described in the methods section. The physicochemical characterization of empty and mRNA-containing LNPs showed very similar size, polydispersity index and zeta potential for both (**Table 3**), indicating that the presence of the mRNA does not greatly modify the structure of the LNP.

**Table 3:** Physicochemical characteristics of the LNPs analyzed with Zetasizer (size, PDI, Zeta potential) and with Qubit (encapsulation efficiency).

Sample	Size (nm)	PDI	Zeta potential (mV)	Encapsulation efficiency (%)
Empty LNPs	162.2 ± 16.0	0.085 ± 0.082	-2.82 ± 0.36	
mRNA-containing LNPs	161.9 ± 2.1	0.100 ± 0.010	-2.59 ± 0.19	91.35 ± 5.25

In addition, the encapsulation efficiency for the mRNA-containing LNPs was calculated at 91.35 ± 5.25% (**Table 3**). The encapsulation efficiency was further tested in agarose gels, demonstrating that the LNPs had a large capacity to enclose and transport the mRNA (**Figure 1d**).

### 3.2 Characterization of the LNPs *in vitro*

**Figure 2:**
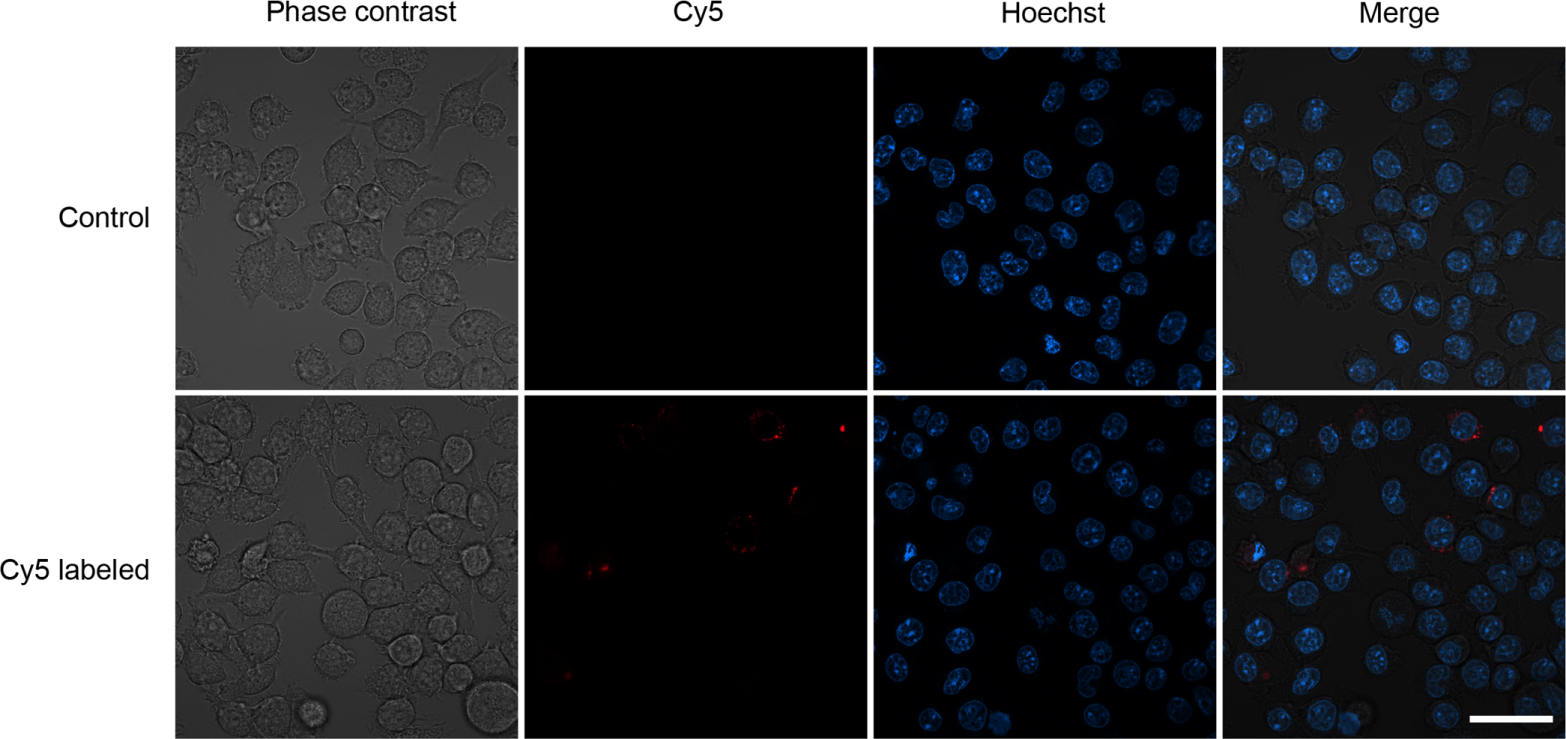
Representative confocal images of LNPs containing unlabeled mRNA (control) or Cy5-labeled mRNA that were added to a culture of RAW 264.7 macrophages. A cytoplasmic red staining can be seen in some macrophages exposed to the labeled LNPs. Scale bar = 20 µm.

To demonstrate that the LNPs were efficiently taken by cells of the innate immune system, RAW 264.7 cells were seeded in microscope-compatible slides and the LNPs, containing either unlabeled (control) or Cy5-labeled mRNA, were added. After an incubation of 2 h, cells were washed and the intracellular presence of labeled LNPs was confirmed (**Figure 2**).

### 3.3 Mouse immunization resulted in increased antibody titers and more CD8^+^ T lymphocytes

**Figure 3:**
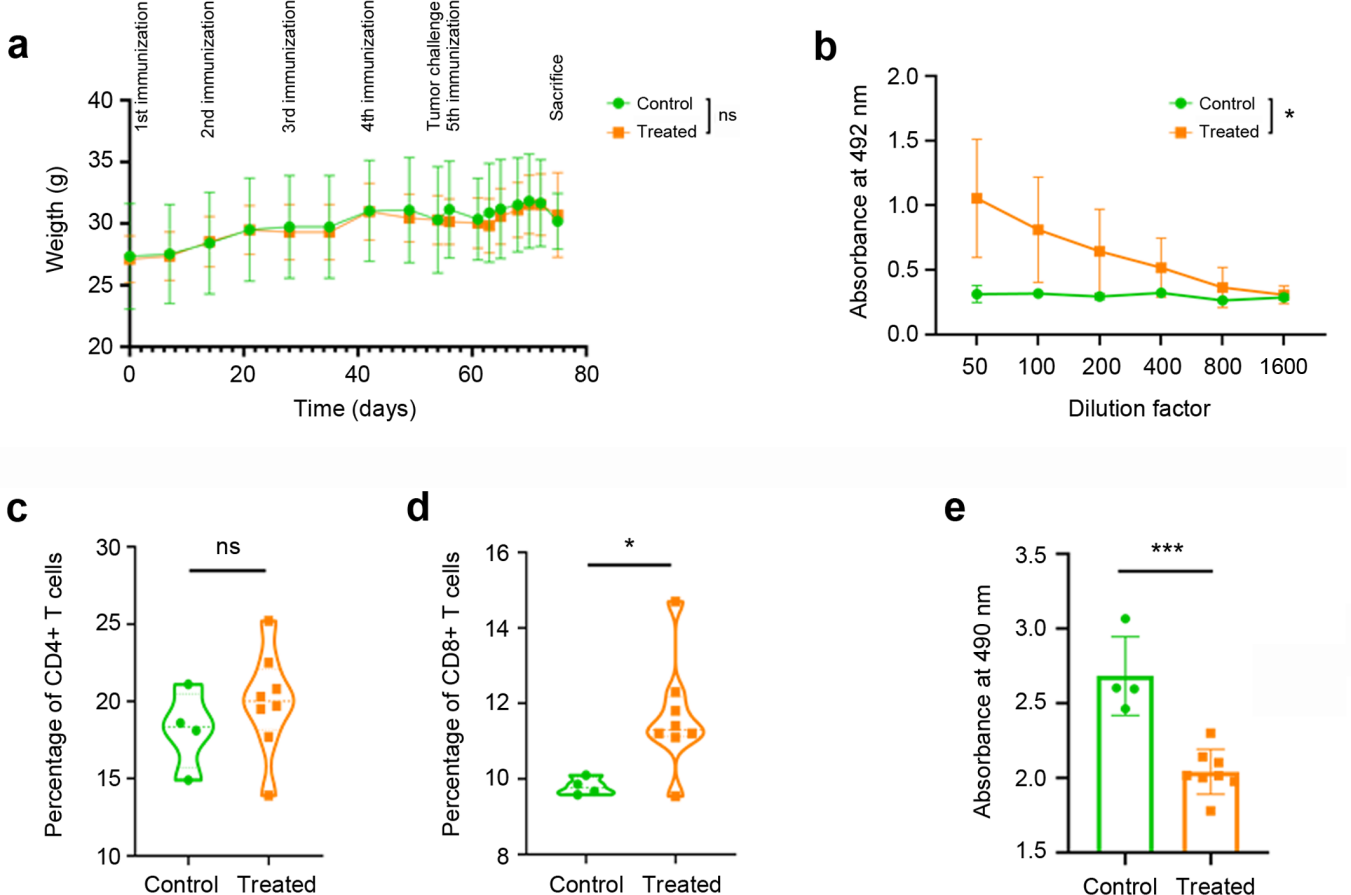
Vaccine results on immune response. Weekly weight of all experimental animals throughout the vaccination process **(a)**. No significant differences were observed between the control animals (green circles) and those receiving the KLH-AM vaccine (orange squares). Humoral immune response in animals receiving empty LNPs (control, green circles) or mRNA-containing LNPs (orange squares), as determined by indirect ELISA **(b)**. Statistical analysis was performed using Two-way ANOVA (*p = 0.040). Data represent mean + SD of all animals in the group (n=10). Cellular immune response was analyzed by flow cytometry using anti-mouse CD4 **(c)** and CD8 antibodies **(d)**. Each point represents the percentage of CD4^+^ or CD8^+^ T cells per 100,000 events per animal. Furthermore, an immune-mediated cytotoxicity assay was performed **(e)**. Statistical analysis was performed using unpaired *t* test (ns: not significant; *p = 0.033; ***p<0.001). Data represent the mean + SD of all animals in the group (n = 4-8).

The KLH-AM vaccine was tested in C57BL/6 immunocompetent mice. During the whole process, mice were weighed weekly to detect any potential toxicity associated to the vaccine. No significant differences in weight were found between the animals receiving the empty LNPs (control) and the mRNA-containing LNPs (**Figure 3a**).

Furthermore, three weeks after the final LNP injection, blood was taken from all animals and the serum was subjected to ELISA analysis in plates coated with synthetic mouse AM. Animals subjected to the KLH-AM vaccine had significantly higher (p=0.040) titles of anti-AM IgGs than those receiving the empty LNPs (**Figure 3b**).

**Figure 4:**
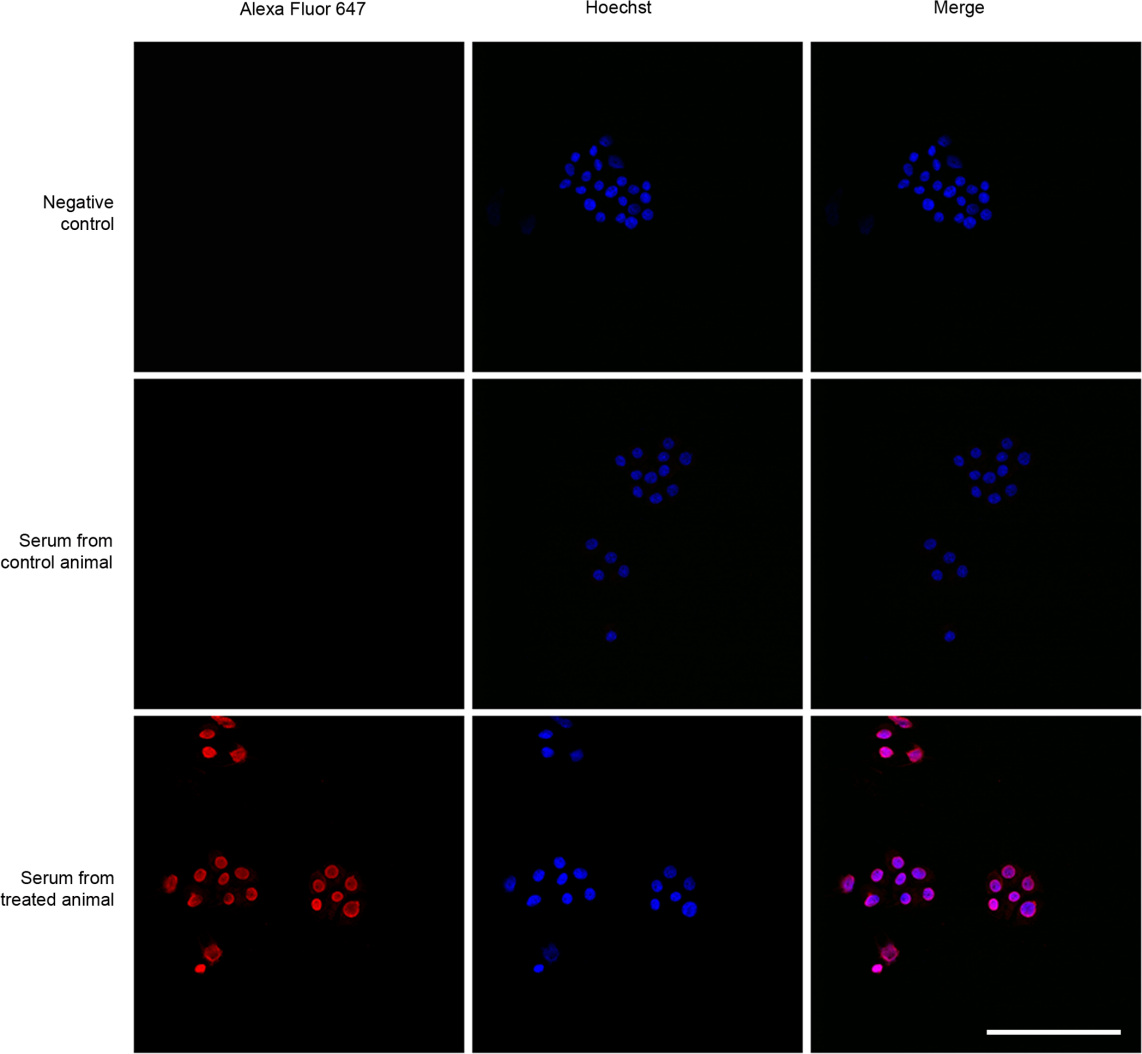
Representative confocal images of cell line PC-12 exposed to sera from vaccinated animals (lower row), sera from control animals (middle row) and PBS as a negative control (upper row). A secondary fluorescently (Alexa Fluor 647) labeled anti-mouse antibody was used to detect bound mouse IgGs. Scale bar = 40 µm.

To further characterize the specificity of the antibodies generated in the vaccinated animals, we used the sera obtained from control and vaccinated mice to stain a cell line, PC-12, that has been shown to produce high levels of AM (35). Only the sera from KLH-AM vaccinated animals was able to produce a strong cytoplasmic signal in the pheochromocytoma cells (**Figure 4**).

After sacrifice, the spleens of some animals were excised and the splenocytes were isolated and analyzed by flow cytometry to study the influence of the vaccine on the contents of CD4^+^ and CD8^+^ lymphocytes (**Supplementary Figure S3**). No significant differences were found between groups for the percentage of CD4^+^ T cells (**Figure 3c**) but there was a significant increase (p = 0.033) of CD8^+^ T cells in the animals vaccinated with KLH-AM LNPs when compared to the controls (**Figure 3d**).

To demonstrate that the splenic T cells had the capability of destroying tumor cells, a co-culture experiment was set up. In each well, 50,000 CD8^+^ T cells were added on top of a culture of 10,000 B16-F10 melanoma cells. These cells were incubated for 30 min, extensively washed to remove floating lymphocytes, and the remaining number of tumor cells was estimated through an MTS assay. The number of tumor cells that were exposed to T cells isolated from KLH-AM vaccinated animals was significantly lower (p<0.001) that those exposed to control T cells (**Figure 3e**).

### 3.4 Mouse immunization resulted in a reduction on the number and size of lung metastases

**Figure 5:**
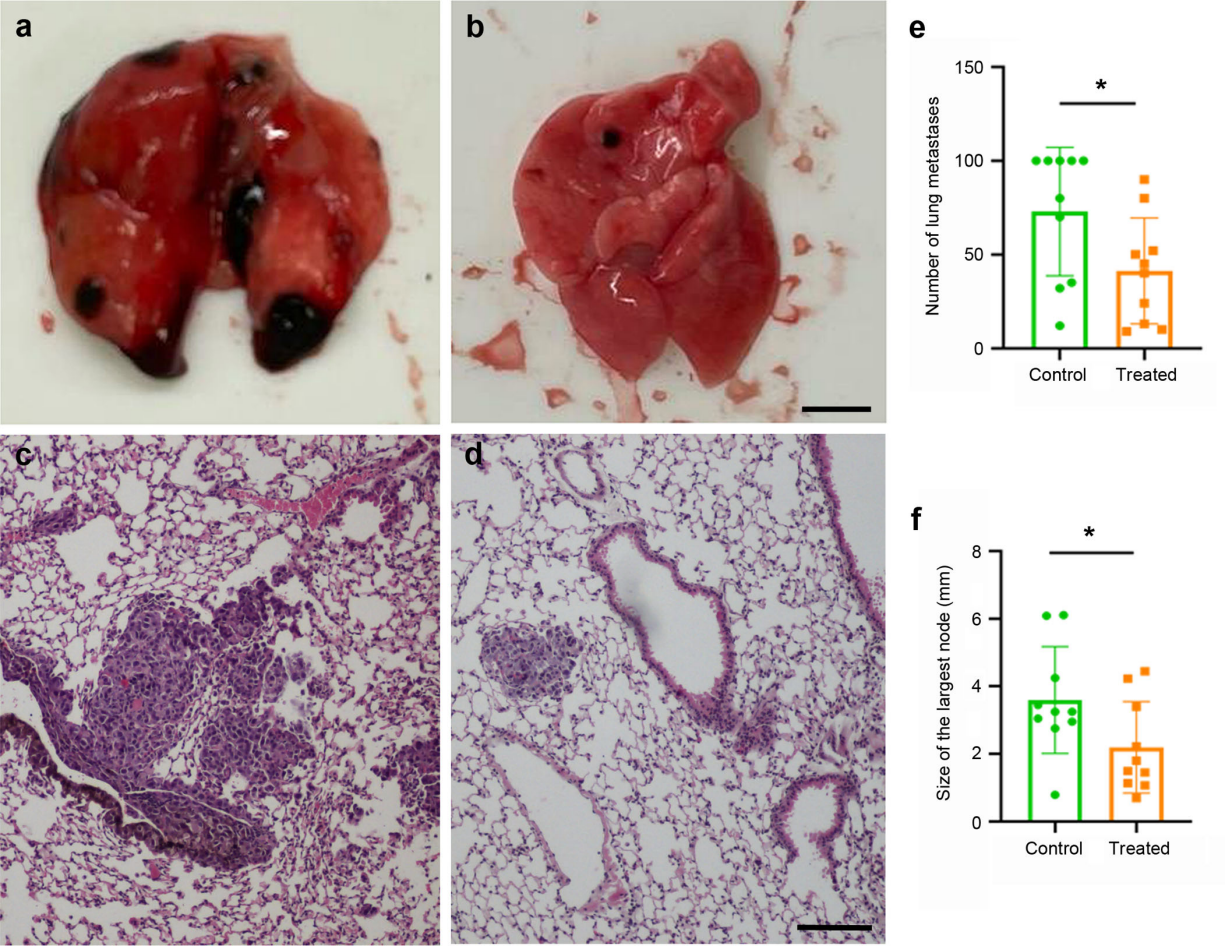
Vaccine effects in tumor growth. Representative macroscopical **(a, b)** and microscopical **(c, d)** images of lungs collected from animals that received empty LNPs **(a, c)** or the KLH-AM LNPs **(b, d)**. The microscopical slides were stained with hematoxylin and eosin. Scale bars: 2.0 mm (a,b) or 100 µm (c,d). Quantification of the number of metastases per lung **(e)** and the size of the largest metastasis in each animal **(f)** in both experimental groups, empty LNPs (green) and KLH-AM LNPs (orange). Statistical analysis performed using unpaired *t* test (*p = 0.028 and 0.020, respectively). Data represent the mean + SD of all animals in the group (n = 10). Some lungs had too many metastases and those were ascribed a value of 100.

After sacrifice, lungs were dissected out and the number and size of the metastases were recorded. Since the B16-F10 cells express high levels of melanin, the metastases were easily visible by the naked eye (**Figures 5a, b**) and were also easily found in histological sections stained with hematoxylin and eosin (**Figures 5c, d**). Just by direct observation, it was clear that mice vaccinated with the KLH-AM LNPs had less and smaller metastases than the animals receiving empty LNPs. In addition, quantification of both the number of metastases (p = 0.028) and the size of the largest metastasis in each animal (p = 0.020) were done and both were statistically significantly lower in animals receiving the KLH-AM vaccine (**Figures 5e, f**).

### 3.5 Mouse immunization resulted in a reduction of tumor angiogenesis

**Figure 6:**
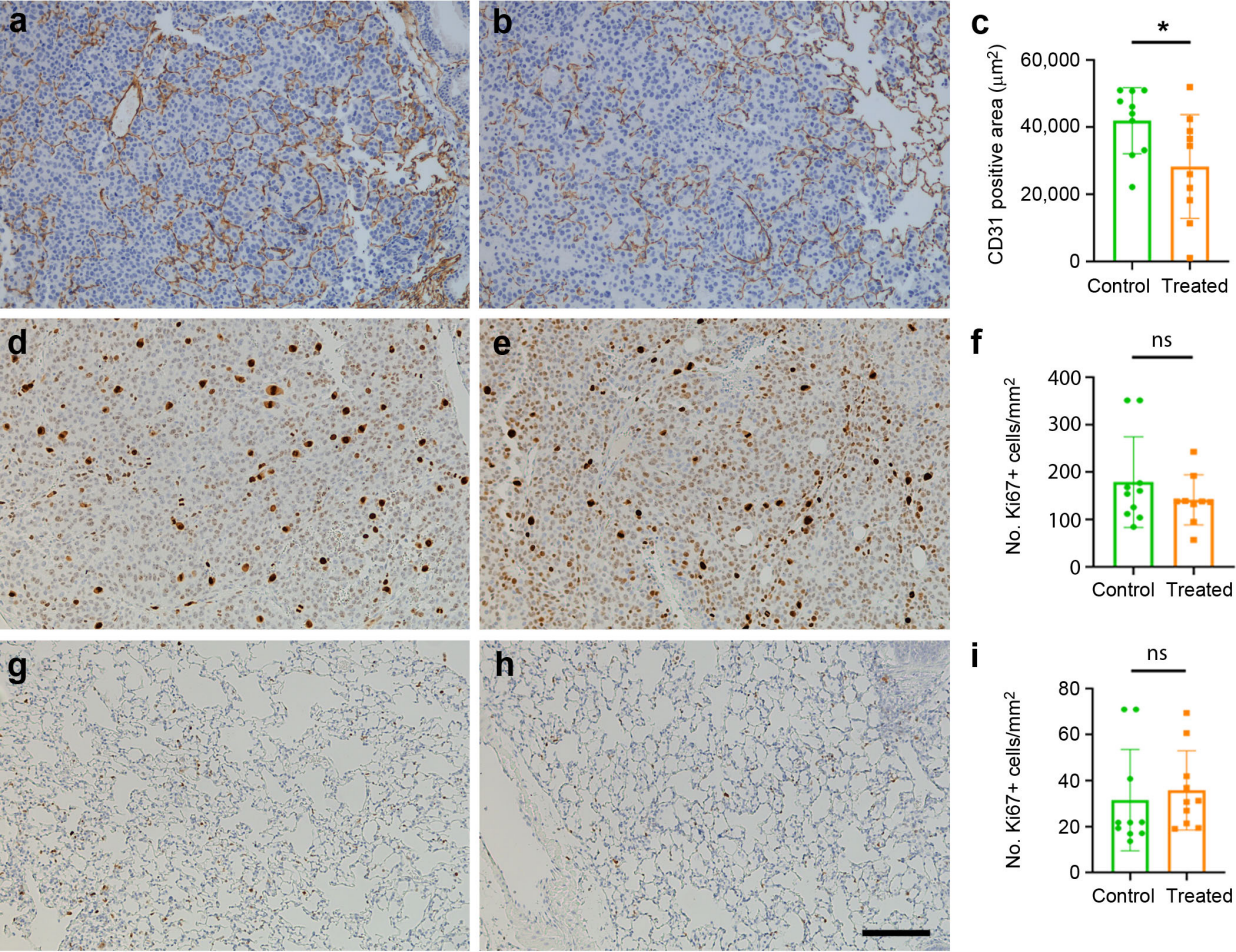
Effects of the vaccine on tumor angiogenesis and cell proliferation. Representative immunohistochemical images of tumor metastases **(a–e)** or lung parenchyma **(g, h)** labeled with antibodies against CD31 **(a, b)** or Ki67 **(d, e, g, h)** in control **(a, d, g)** and in mice treated with KLH-AM LNPs **(b, e, h)**. Quantification of the area occupied by the positive vessels **(c)** shows significant differences between groups. Statistical analysis was done with Mann-Whitney-U test (*p = 0.046). In contrast, there was no significant (ns) difference in the number of Ki67^+^ cells either in the metastases **(f)** or in the lung parenchyma **(i)**. Data represent mean + SD of all animals in the group (n=10). Scale bar = 150 µm.

Since the goal of this study was to target an angiogenic peptide, we also studied the area occupied by CD31^+^ vessels within the tumors. Within the metastases, a significant (p= 0.046) reduction in the area covered by CD31^+^ immunoreactive vessels was found in mice vaccinated with the KLH-AM LNPs when compared with the empty LNPs (**Figures 6a–c**).

### 3.6 Mouse immunization did not influence the proliferation index of tumor or parenchymal cells

Since AM has been characterized as an autocrine growth factor for tumor cells (38), we investigated whether the vaccine had an impact on the proliferation index of the tumors by using an antibody against Ki67. No significant changes were found between groups (**Figures 6d–i**).

### 3.7 Mouse immunization did not compromise existing blood vessels

**Figure 7:**
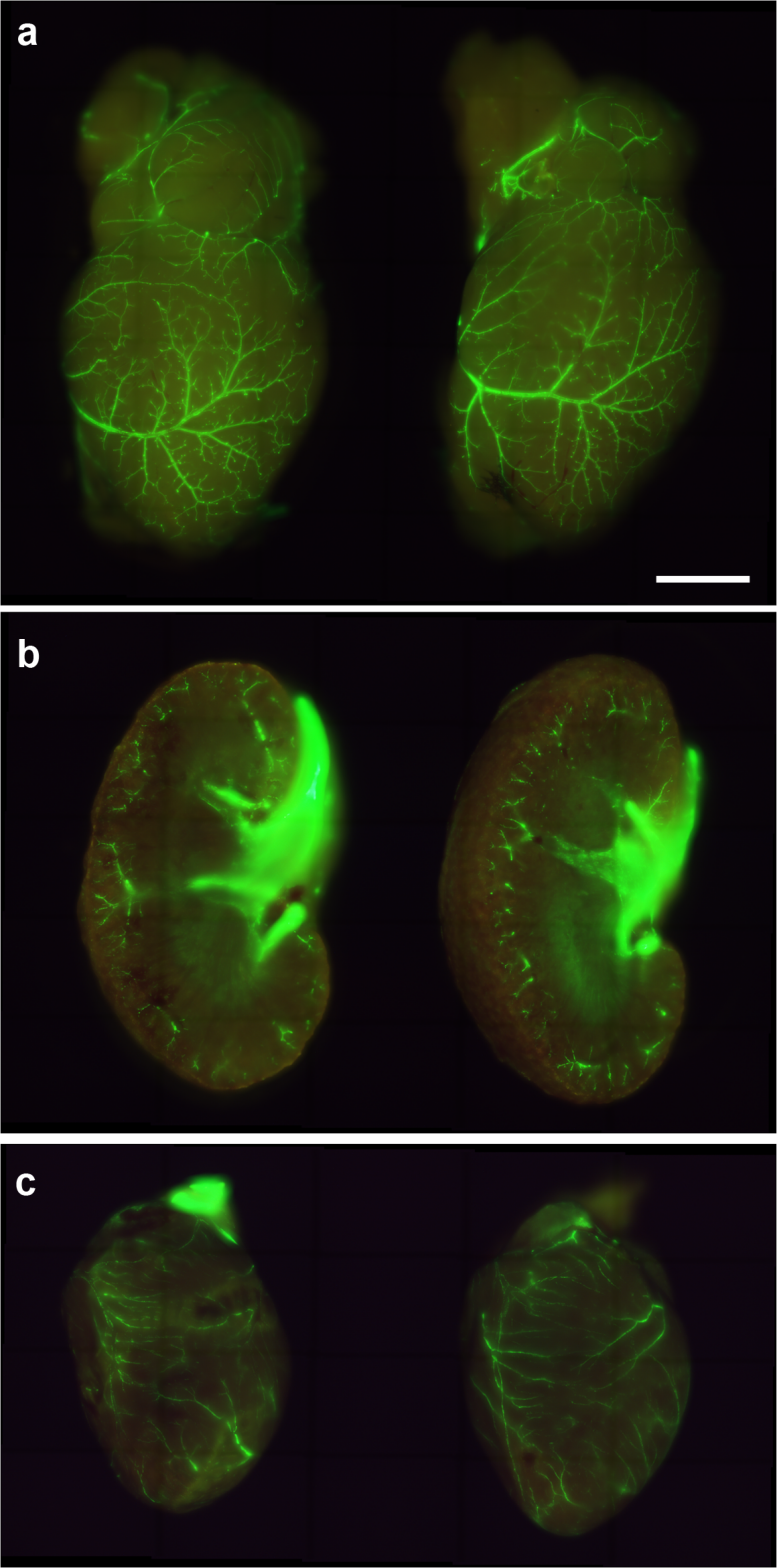
Representative images of the brain **(a)**, kidney **(b)** and heart **(c)** of SMAA-GFP mice that received either the control (left column) or the KLH-AM LNPs (right column). Scale bar = 2.0 mm.

After immunization of SMAA-GFP animals with either the empty or the KLH-AM LNPs, tissues with a high number of blood vessels were analyzed under UV light (**Figure 7**). As can be appreciated in the images, no differences were found in the number of vessels or their distribution between both experimental groups.

## 4 Discussion

In this study, we have designed, built and characterized an RNA vaccine targeting the angiogenic peptide, AM. The vaccine was able to induce humoral and cellular immune responses and resulted in the reduction of angiogenesis and tumor burden in a melanoma lung metastasis model. Furthermore, using transgenic mice with fluorescent blood vessels, we showed that the vaccine had no impact on existing vasculature.

AM is a proangiogenic factor but it influences tumor cell biology in many other ways, including the promotion of tumor cell growth, migration, metastasis and escape from immune surveillance (17), making it a very exciting target for cancer therapy. Therefore, the antitumor effects we see in vaccinated animals may be a combination of all these pathophysiological actions of AM rather than a pure antiangiogenic response. Nevertheless, we did not find changes in the proliferation index of the tumor cells, although AM has been described as an autocrine growth factor for many tumor cells (38). This fact suggests that the main functions of AM on these melanoma models is inducing angiogenesis and dampening the immune response. Several approaches have been taken to try to reduce AM´s activity in the context of cancer therapy. They include the use of monoclonal and/or polyclonal antibodies against either the peptide (38) or its receptors (39), small molecules that inhibit AM´s actions (40), use of carboxy-terminal peptide fragments (41), or siRNA-based molecular strategies (42), among others. Nevertheless, to the best of our knowledge, no vaccines targeting AM are available in the literature.

The immune response elicited by the AM vaccine was both humoral and cellular. On the one hand, there was a significant increase of anti-AM antibody titers in the vaccinated animals. The specificity of those vaccine-induced antibodies was confirmed by staining PC12 cells that have been previously shown to express high levels of AM (35). As for the cellular response, a significant increase of CD8^+^ T cells was found in the animals receiving the KLH-AM LNPs. The presence of AM-directed cytotoxic T cells was demonstrated by co-culturing melanoma cells with T cells isolated from the spleens of vaccinated animals and showing a significant decrease of the former after 30 min of culture. It has been shown that tumor cells present AM binding sites in their membrane and that AM acts as an autocrine growth factor for these cells (38). Therefore, we can envision the presence of AM bound to its receptor on the surface of the tumor cells, offering a ready target for the AM-activated cytotoxic T cells, resulting in the destruction of said tumor cells.

Another way for AM to contribute to cancer cell growth is to enhance escape from immune surveillance. This is accomplished by several mechanisms. One of them includes a direct modulation of the adaptive immune cells. For instance, T cells and dendritic cells have been shown to have AM receptors in their membrane (43). Exposure to AM induces production of regulatory T cells (T_reg_) (44), and a partial maturation of dendritic cells (45), all of these contributing to a more tolerogenic environment within the tumor. Thus, it is not surprising that a vaccine against AM should increase immune-mediated antitumor effects.

Another mechanism of immune surveillance escape is caused by AM´s affinity for its binding protein, complement factor H (46). In this binding, each partner enhances the function of the other, and since complement factor H is the main inhibitor of the alternative pathway of the complement cascade, the presence of AM increases the blocking of this mechanism of the innate immune system. It has been shown that tumor cells have binding sites for both complement factor H and AM, and that the presence of these molecules effectively blocks complement-mediated cytotoxicity (47). Therefore, a vaccine directed against AM may also indirectly contribute to the demise of tumor cells by reactivating complement-mediated mechanisms.

A general criticism on antiangiogenic therapies is that they may induce unwanted side effects by interfering with normal angiogenesis and/or existing blood vessel stability. Fortunately, angiogenic processes are relatively limited in adult and elderly cancer patients but, obviously, such therapies should be avoided in pregnant women and in developing children, where angiogenesis is rampant. Interestingly, many studies on antiangiogenic DNA vaccines have shown a complete lack of these side effects (48, 49), demonstrating that vaccine blockade of angiogenesis does not necessarily implies problems in wound repair and/or reproduction. This may be due to the fact that tumor angiogenesis often produces rather disorganized, imperfect, and unstable blood vessels (50), which may be the main targets for the vaccines whereas the physiologically well-organized angiogenic process involved in wound healing or preexisting vessels may not be affected by them. In any case, there were still other studies that found a delayed wound healing in vaccinated animals but no effects on fertility (51, 52). Therefore, caution should be maintained with any new antiangiogenic vaccine product. In our case, we found no changes in existing vasculature but wound healing and fertility tests need to be performed in the future.

Due to the fast growth of the syngeneic B16-F10 tumor models, this study was performed as a prophylactic vaccination strategy, where mice were first immunized and then they received the tumor challenge. Obviously, this approach will not be feasible in a clinical setting for the general population and we envision this vaccine either as a therapeutic option for patients with active cancer disease, or as a preventative approach for patients that have been already treated for a primary tumor and are therefore at high risk of developing cancer recurrence (53). For instance, the rate of breast cancer recurrence is 14.4% (54) and the appearance of a second primary cancer is 23.0% for oral squamous cell carcinoma (55). Of course, this vaccine could be used by itself, but a more realistic view is to think about its combination with additional approaches including chemo-, radio-, or immune-therapies, which is the current approach for most antiangiogenic drugs (56).

This study has some limitations. First, this was a proof-of-concept study and we used only males to avoid the interference of the estrous cycle. Nevertheless, it is well known that men and women are differently affected by cancer. Part of these differences may be related to diverse risk behaviors (tobacco, sun exposure, diet, physical exercise, etc.), but genetic and physiological predisposition must be taken into consideration (57). For instance, the different hormone levels may influence tumor cell growth and metastasis. Therefore, future studies will address this issue vaccinating both male and female mice. In addition, testing whether the vaccine can prolong survival with the proper experimental design should be very relevant for its potential applicability. Another limitation of this manuscript is that we did not perform wound healing experiments and, although we did not see changes in pre-existing vessels, we cannot rule out that our vaccine may interfere with this physiological angiogenesis process, which would be important for potential patients, especially if they need to undergo surgery. Furthermore, a deeper characterization of the immune cells involved in the vaccine´s response will offer a better understanding of the process.

In summary, we have shown that it is possible to generate an mRNA vaccine against an intrinsic angiogenic peptide, with promising anticancer results and low toxicity. A clear trend in mRNA cancer vaccine development is the encapsulation of different mRNAs into the same LNP, simultaneously targeting diverse antigens. This allows a personalized approach since those neoantigens are chosen from the tumor molecular landscape of the patient. For instance, BioNTech is testing one of these vaccines, containing 20 mRNAs, named “autogene cevumeran”, in pancreatic ductal adenocarcinoma patients (58) and other solid tumors. Another mRNA vaccine developed by Moderna and Merck (mRNA1457/V940) targets 34 different neoantigens and has achieved impressive results in a phase 2b clinical trial with advanced melanoma patients (59) and is currently undergoing a phase 3 trial. Looking at our results, we suggest that including at least an angiogenic factor among the other targets in these multiplexed vaccines may result in a great advantage for cancer patients.

## Data Availability

The original contributions presented in the study are included in the article/**Supplementary Material**. Further inquiries can be directed to the corresponding author.
