# Supplementary material for: An RNA vaccine against adrenomedullin reduces angiogenesis and tumor burden in a syngeneic metastatic melanoma mouse model
Source: Front Immunol. 2025 Jun 5;16:1604156. doi: 10.3389/fimmu.2025.1604156 (PMC12176902; doi:10.3389/fimmu.2025.1604156)
Supplement: Supplementary file 1 [file Table1.docx]

Supplementary Material

# Selection of the optimal KLH fragment

To find the KLH1 sequence with the most promising adjuvant features and reduce the size and complexity of the final sequence, we analyzed each of eight KLH1 functional units on IEDB. This database provides information based on physicochemical properties of the sequence analyzed and it correlates with empirical laws and data generated in the literature. The scores for each FU, for B cell linear epitope binding prediction properties, including presence of linear epitopes, immunogenicity, beta turn, flexibility, hydrophilicity and surface accessibility were compared and it was observed that FUg achieved the highest scores, in average (Fig. S1a). The same was observed when 3D binding of B cell epitope capacity was assessed with DiscoTop thus further supporting high probability that this sequence can initiate a humoral immune response (Fig. S1e). Furthermore, binding and immunogenicity prediction for MHC I epitopes for several mouse alleles including H2-Db, H2-Dd, H2-Kb, H2-Kd, H2-Kk and H2-Ld, were made and analyzed. Results were more complex considering that, based on different alleles, different sequences were predominant. However, on average FUg was the sequence being dominant for several alleles both for binding and immunogenicity predictions (Fig. S1b,c). Similar results were observed for MHC II binding prediction (Fig. S1d).

The FUg sequence was further analyzed and 7 parts of the sequence were compared for both B cell epitope and T cell epitope properties predictions. For B cell epitope, both linear and 3D binding and immunogenicity predictions, considering the short length of individual sequence parts, the analysis was performed by manually marking the sequence area with the highest scores (red rectangles, Figure S2a-c). For MHC I and II epitope binding and immunogenicity predictions, analysis was performed as before mentioned (Figure S2d-f).

In average, sequence parts 2 and 3, ranging from 60 bp to 180 bp, were the ones that achieved highest scores in average in both analyses and thus these were used in the construction of the final sequence.

**
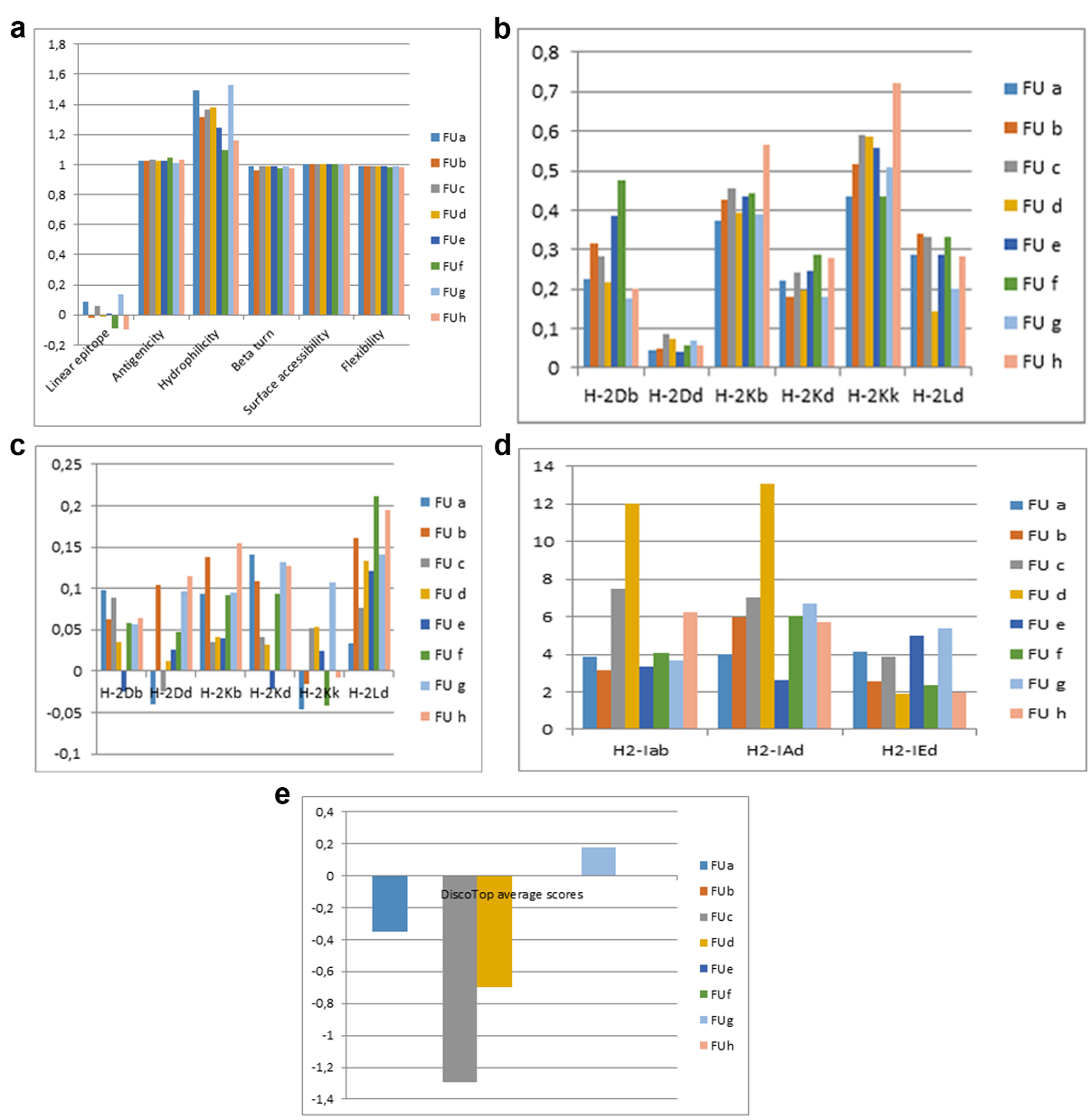
**

**Supplementary Figure S1**. Analysis and comparison of each KLH1 functional unit. a) Linear B cell epitope analysis; b) MHC I binding analysis for each mouse allele; c) MHC I immunogenicity analysis for each mouse allele; d) MHC II binding analysis for each mouse allele; and e) Discontinuous B cell epitope analysis. Results are represented as scores for a,b,c and e while, for d, results are represented as adjusted ranks.


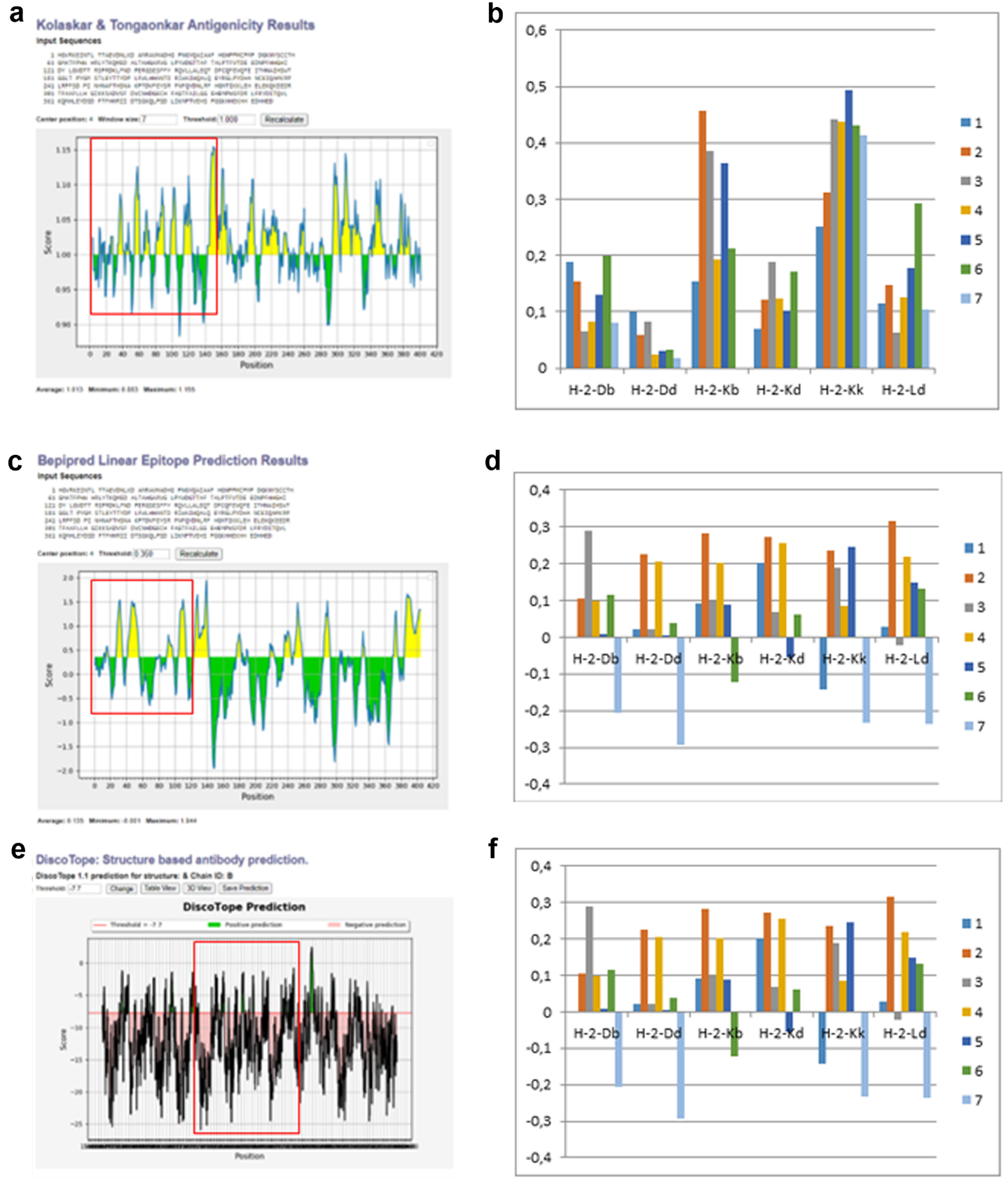


**Supplementary Figure S2**. Analysis and comparison of different parts of KLH1 FUg sequence. Figures on the left (**a,c,e**) show computer screen captures during the analysis. Figures on the right (**b,d,f**) show actual values. (**a,b**) MHC I binding analysis for each mouse allele; (**c,d**) MHC I immunogenicity analysis for each mouse allele; (**e,f**) MHC II binding analysis for each mouse allele. Sequence 1 = 1-60 bp, 2 = 60-120 bp, 3= 120-180 bp, 4 = 180-240 bp, 5 = 240-300 bp, 6 = 300-360 bp, 7 = 360-404 bp. MHC I binding and immunogenicity prediction results are represented as scores, while MHC II binding prediction results are represented as adjusted ranks.


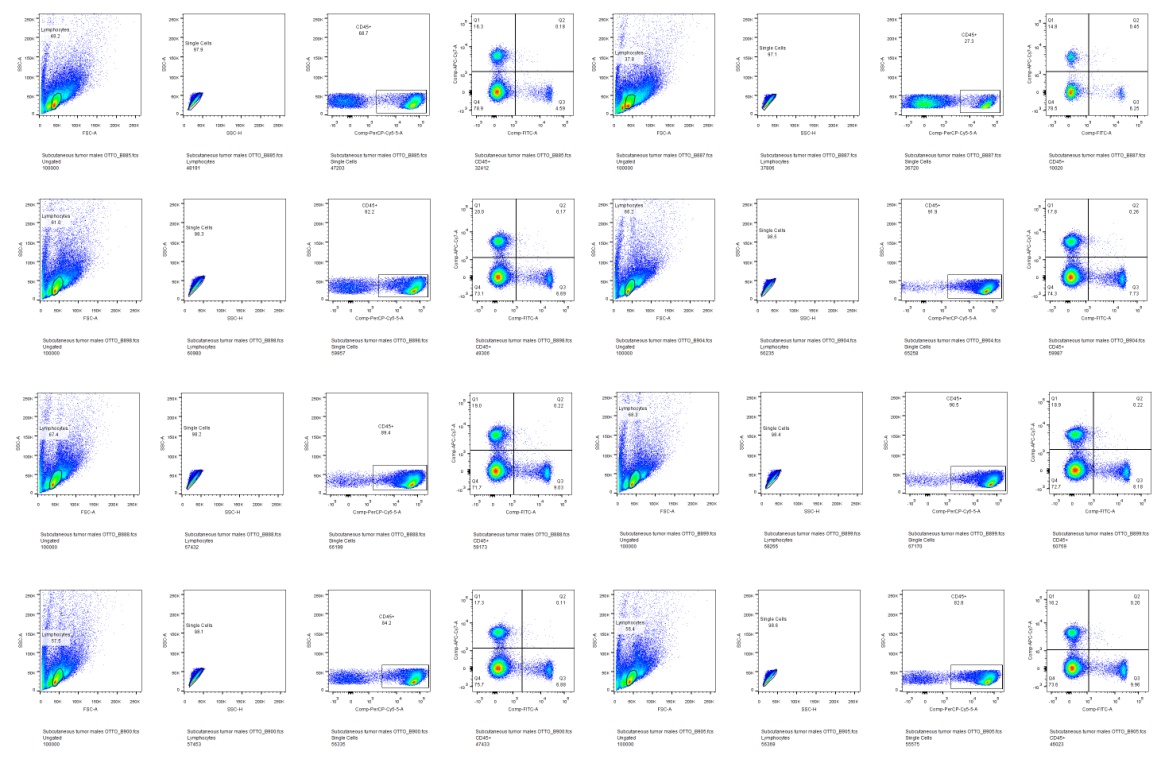


**Supplementary Figure S3**. Representative flow cytometry results to study the cellular immune response using anti- mouse CD45, CD4 and CD8 antibodies. The gating strategy was applied using FlowJo software and this layout shows representative results from control (n=4) and treated (n=4) animals. The gating strategy included gating on lymphocyte population based on forward- (FSC) and side- (SSC) scatter and then separating single cells population by using SSC area and height. Furthermore, gating included separation of CD45^+^ cells and then, from this subpopulation, separation of CD4^+^ and CD8^+^ cells. The compensation controls were prepared for each experiment by using single stained and unstained cells.
